# Symmetric Lipomatosis Arising in the Tongue Presenting as Macroglossia and Articulatory Disorder

**DOI:** 10.1155/2016/2061649

**Published:** 2016-06-19

**Authors:** Masanori Kudoh, Ken Omura, Arata Satsukawa, Koshi Matsumoto, Takahide Taguchi, Hiroyuki Harada, Yoshimasa Ishii

**Affiliations:** ^1^Division of Oral and Maxillofacial Surgery, Ebina General Hospital, 1320 Kawaraguchi, Ebina, Kanagawa 243-0433, Japan; ^2^Oral and Maxillofacial Surgery, Department of Oral Restitution, Division of Oral Health Sciences, Graduate School, Tokyo Medical and Dental University, Tokyo, Japan; ^3^Division of Oral and Maxillofacial Surgery, General Tokyo Hospital, Tokyo, Japan; ^4^Division of Diagnostic Pathology, Ebina General Hospital, Ebina, Japan

## Abstract

Symmetric lipomatosis is definitively characterized by symmetric, tumorous lipomatous proliferation of adipose tissue that often develops in the head and neck, shoulders, and upper trunk. However, in the oral region, symmetric lipomatosis of the tongue (SLT) is an extremely rare condition related to generalized lipidosis that is often caused by chronic alcoholism. It is characterized by multiple symmetric lipomatous nodules and diffuse bilateral swelling located within the tongue. We report an extremely rare case of SLT arising in an 80-year-old man with a long history of alcoholic liver cirrhosis. He exhibited multiple soft nodular protrusions on the bilateral margin of the tongue presenting as macroglossia for years. Although MR imaging showed multiple fatty masses on both sides of the tongue, there was no elevated tumor mass on the bilateral margin. The patient underwent bilateral partial glossectomy under general anesthesia. Histopathologically, the resected tumor exhibited diffuse infiltration with mature adipose tissue lacking a fibrous capsule. Due to the lipidosis and the unusual presentation of multiple lesions, the lesion was ultimately diagnosed as SLT. At present, after surgery, the patient wears a full-denture and is in excellent condition, with no sign of recurrence, improved QOL, and recovery of masticatory, articulatory, and speech intelligibility functions.

## 1. Introduction

Benign symmetric lipomatosis (BSL) was first reported by Brodie in 1846 [1]. This disease develops in the head and neck, shoulders, and upper trunk. However, it is extremely rare arising in the oral region bilateral side. BLS was characterized by diffuse symmetric deposit of adipose tissues in the neck. On the other hand, symmetric lipomatosis of the tongue (SLT) is a rare disease. SLT was first reported by Desmond [2] in 1944, a symmetrical and diffuse bilateral swelling at the border of the tongue. Ogawa et al. [3] have suggested that SLT may be a subtype of BSL, as both conditions share common pathological features. Calvo-García et al. [4] reported that BSL is common in Mediterranean people; in 6 cases of SLT, all patients were male and mean age was 67 years (61–71 years). However, SLT is more frequent in the Japanese population but rarely develops bilateral margins of the tongue, with only 2 previous case reports of this condition [2, 4]. Herein, we reported an extremely rare case of SLT presenting as macroglossia and articulatory disorder.

## 2. Case Report

**Figure 1:**
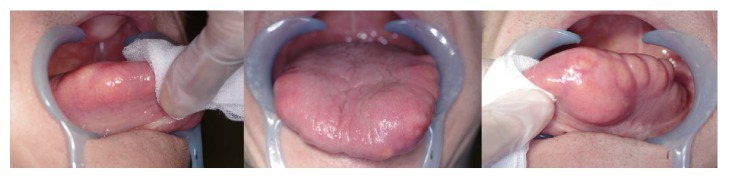
Intraoral findings at the first visit. Multilocular tumors were found on the bilateral margins of the tongue as macroglossia.

The patient was an 80-year-old man who was referred to our division for the chief complaints of macroglossia and articulatory disorder. Oral findings showed painless tumors on the bilateral margins of the tongue and dysarthria/dysphasia (Figure 1). His medical history included alcoholic liver injury, dyslipidemia, hypertension (HT), chronic gastritis, diabetes mellitus (DM), acute pancreatitis, glaucoma, and dementia. The patient was a heavy drinker. He had felt discomfort in the bilateral tongue margins for several decades but left it untreated. During an upper gastrointestinal endoscopy in the nursing home, tumors were found on the bilateral tongue margins and he was referred to our department. Findings at the first visit included a poor nutritional status (height: 160.0 cm, body weight: 52.7 kg, and BMI: 20.0 kg/m^2^). Extraoral findings showed facial symmetry and a good complexion. There were no significant findings in the cervical lymph nodes. Preoperative laboratory examination revealed high levels of TG (202 mg/dL), but TC and HDL and LDL-cholesterol were normal.

Multilocular tumors were found on the bilateral margins of the tongue, which formed macroglossia, and xanthic, elastic soft tumors were evident in the shallow submucosa. The surfaces of the tumors were smooth, and in particular, the left tongue margin was significantly elevated.

**Figure 2:**
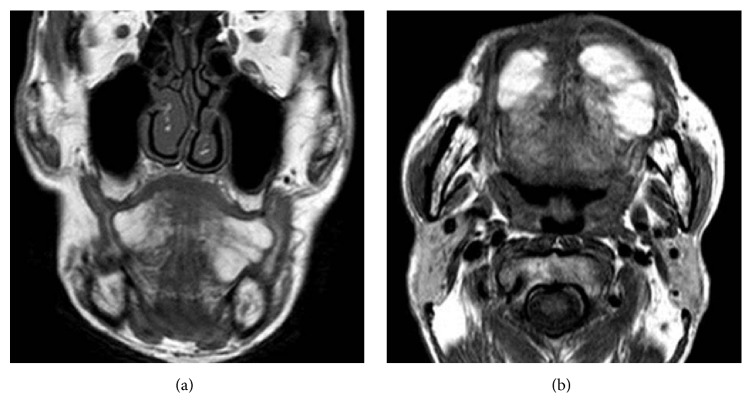
MR images ((a) axial view, (b) horizontal view). MR imaging shows high-intensity nodules and adipose tissue invading into the bilateral lingual muscle.

**Figure 3:**
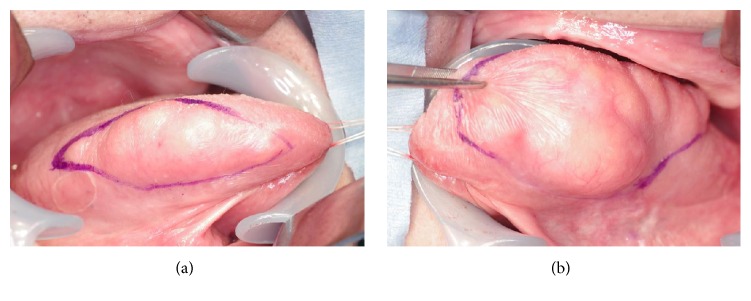
Intraoperative view. Surgical incision ((a) right side, (b) left side).

**Figure 4:**
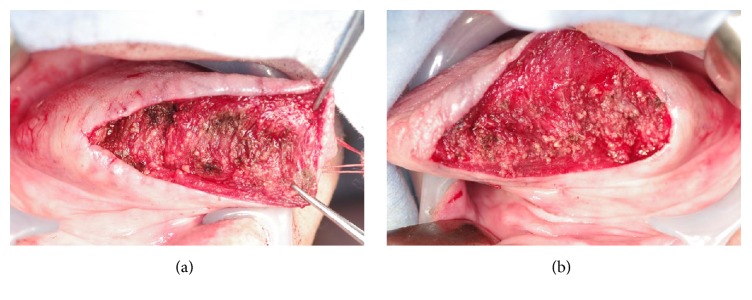
Intraoperative view. After bilateral partial glossectomy (tumor excision).

While there were no abnormalities in perception/taste, dysarthria was observed. MR images showed T2 hyperintensity and well-defined borders in the bilateral margins of the tongue and ill-defined trabecular structures like muscle bundles in the deep part of the tongue, which suggested that it might be symmetric lipomatosis (Figure 2). The patient underwent bilateral partial glossectomy (tumor excision of the tongue) under general anesthesia. A spindle-shaped incision was made along the base of the tumors and the mucosa was detached along the tumor on the tongue margins (Figure 3). The tumor margin was not clear in the deep portion and the tumor partially invaded into the muscle layer. Considering the potential for postoperative dysfunction, the muscle layer was also removed en bloc with the tumors (Figure 4). While there were finely granular, lipoma-like lesions on the resected surface, conservative treatment was applied as resection of the abovementioned lesions might induce dysfunction. After confirming hemostasis, the incision was sutured and closed.

**Figure 5:**
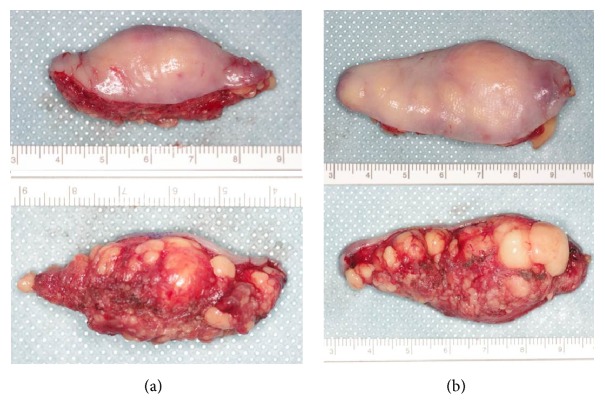
Surgical specimen ((a) right side, (b) left side). The resected specimens showed a relatively well-defined border in the mucosa but the encapsulation was not clear in the deep portion.

The resected specimens showed a relatively well-defined border in the mucosa but the encapsulation was not clear in the deep portion (Figure 5).

**Figure 6:**
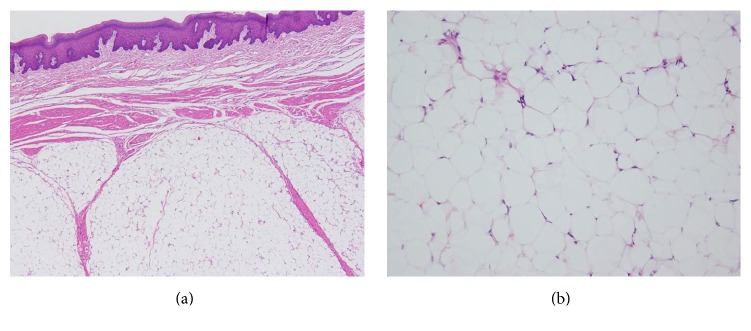
Histopathological image ((a) H-E ×100, (b) H-E ×200). The tumor consisted of proliferation of mature adipocytes.

The tumor consisted of proliferation of mature adipocytes, and there were no signs of encapsulation in the muscle layer or atypia, such as mitotic figures (Figures 6(a) and 6(b)). Based on the abovementioned findings, the histopathological diagnosis showed lipomatosis. Therefore, clinically, the final diagnosis was SLT presenting as macroglossia.

**Figure 7:**
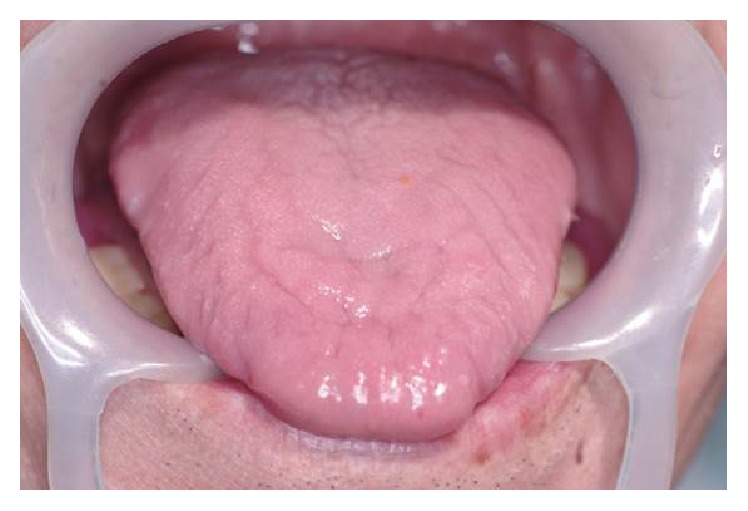
Postoperative intraoral view. At present, after surgery, the patient is in excellent condition, without recurrence and with improved QOL.

At the present time, there has been no sign of postoperative dysfunction or complication, and the dysarthria and dysphasia have improved significantly. The patient's clinical course remains excellent (Figure 7).

## 3. Discussion

Lipoma is the most common nonepithelial benign soft tissue neoplasm and monostotic solitary lesion and can occur in any part of the body. Meanwhile lipomatosis is one of multiple systemic tumors that are characterized by diffuse proliferation of adipose tissue in normal tissues. Lipomatosis is differentiated from lipoma by specific characteristics, such as ill-defined borders and nonencapsulation. However, while lipomatosis is relatively rare in the oral region, it can sometimes develop bilaterally on the tongue.

Lipomatosis in the tongue is classified by gross appearance into two types, nodular type and diffuse type [5]. The nodular type is defined as multiple masses with diameter of a few mm to several mm that develop bilaterally, whereas the diffuse type exhibits a flat surface and symmetrical diffuse swelling that spreads in the form of a band [6]. In our case, multiple nodular tumors consisting of proliferation of mature adipocytes were observed bilaterally. According to the definition of Enzinger et al. [6], it was considered to be symmetric lipomatosis (nodular type). There are several predisposing factors that can be systematically or locally derived. The systematic factors include congenital causes, genetic factors, endocrine dysfunction, lipidosis caused by heavy use of alcohol, neuropathy, trauma, tuberculosis, and radiation [6]. In the process of lipid metabolism, apolipoproteins bind lipids to form lipoproteins that circulate in the blood stream; low-density lipoprotein (LDL), one of the lipoproteins, is taken up by extrahepatic tissues via receptors where they are degraded and taken up. Some abnormalities may occur during these metabolic processes and lipid may be deposited on the tongue [7]. Furthermore, as the tumor rarely develops bilaterally in regions other than the oral cavity, abnormal anatomical structure of the tongue or stimulation and trauma by teeth/occlusion are also suspected as being causative [8].

With respect to the association between SLT and alcoholic liver injury, fatty deposits in the body might occur due to increased blood acetyl CoA levels, which is the primary source of fatty acid derivatives during the metabolism of alcohol to acetate by alcohol dehydrogenase and acetaldehyde dehydrogenase in the liver [9, 10].

The most common causes are alcohol dyslipidemia and liver damage, as in the present case, followed by HT, viral hepatitis, and stroke. The local factors include fatty degeneration with aging of the superficial lingual muscle, hyperplasia of the fat layer of the lingual septum, adipose tissue formation during granulation formation caused by occlusion error, and stimulation and trauma caused by the teeth/occlusion. In our case, we considered that the systemic factor was alcohol dyslipidemia and that the local factors were fatty degeneration with aging of the superficial lingual muscle, which was proposed by Staudt et al. [10], and hyperplasia of the fat layer of the lingual septum. SLT occurs more commonly in the elderly and only in men. There are 2 types, the nodular type and the diffuse type. In our case, the nodular type-multiple lipomatosis could become larger and aggregate, which might ultimately develop into the diffuse type.

Recently, there have been some reports of treatment strategies, such as tumor resection and surgical debulking, with consideration also given to prevention of dysfunction [5]. Recently, Tohya et al. suggest that the surgical strategies of SLT showed improvement of the articulatory and speech intelligibility disorder and dysphagia. SLT is not encapsulated but invades into the deep tissue, which suggests that any residual mass could induce recurrence. The presence of dysfunction and/or systemic illness such as alcoholic liver injury and hepatic cirrhosis after radical glossectomy strongly suggests the presence of new emerging lesions and the significance of long-term close follow-up, as well as continuous dietary and life guidance. His postoperative course is excellent at present. The reports of recurrence [5] after resection and malignant transformation [11] from lipomatosis (development of liposarcoma) suggest the need for long-term close follow-up.
